# Risk of pedicle and spinous process violation during cortical bone trajectory screw placement in the lumbar spine

**DOI:** 10.1186/s12891-020-03535-4

**Published:** 2020-08-11

**Authors:** Lilian Zhang, Naifeng Tian, Jian Yang, Wenfei Ni, Liya Jin

**Affiliations:** Department of Orthopaedic Surgery, The Second Affiliated Hospital and Yuying Children’s Hospital of Wenzhou Medical University, 109 Xueyuanxi Road, Wenzhou, 325000 Zhejiang China

**Keywords:** Cortical bone trajectory. Spinous process violation. Screw penetration. Three-dimensional simulation

## Abstract

**Background:**

Previous studies have confirmed the feasibility of the cortical bone trajectory (CBT) technique. However, there are few reports on spinous process violation and screw penetration during the screw insertion. The purpose of this study was to evaluate the incidence of spinous process violation and screw penetration through the pedicle during CBT screw insertion.

**Methods:**

Computed tomography (CT) scans with normal lumbar structures were consecutively obtained and three-dimensional (3D) reconstructions of the lumbar spine were created. Bilateral CBT screw placement was simulated on each segment using a screw diameter of 4.5 mm, 5.0 mm, or 5.5 mm. Incidences of these complications were recorded and analyzed.

**Results:**

A total of 90 patients were enrolled. Spinous process violation was observed in 68.3, 53.3, 25.5, 1.7, and 0% from L1 to L5, respectively, using 4.5 mm screws. A significant difference was found among the five segments but this was unconnected to gender or screw diameter. The incidence of screw penetration through the inner wall decreased from L1 to L4; in turn, L1 (16.7–35.5%), L2 (12.7–34.4%), L3 (2.8–23.8%) and L4 (1.1–6.7%). This trend was reversed in L5 (6.7–16.7%). Moreover, screw penetration through the outer wall was rare. The incidence of screw penetration varied with screw size as well as lumbar level, but not with gender.

**Conclusions:**

There are more difficulties of CBT screw fixation in upper lumbar spine. The low rate of screw penetration, using 4.5 mm screws, suggests the safety for CBT fixation in the lumbar spine. Larger screws (5.0 mm or 5.5 mm) are more recommended for use in the lower lumbar spine. Moreover, CBT fixation in L5 deserves greater attention because of the unique morphology of the pedicle.

## Background

The pedicle screw fixation technique is widely used as an effective surgical method for spinal segmental fixation. Traditional pedicle screw fixation has been considered to be the optimal method for it has high level of stability. However, this technique is invasive and requires significant soft tissue dissection. Moreover, it should be noted that screw loosening or dislocation is a common problem in traditional trajectory surgery in elderly patients with severe osteoporosis [1–3]. To overcome these deficiencies, Santoni et al. [4] suggested the cortical bone trajectory (CBT) screw fixation technique as an alternative strategy for rigid fixation in the lumbar spine. CBT screws follow a specific trajectory from the pedicle to the cortical bone surface, and improvement in screw purchase and reduction of the loosening rate has been confirmed [5, 6]. To date, this technique has been applied clinically as an alternative fixation method in osteoporosis patients, and has demonstrated satisfying clinical outcomes [7, 8]. Moreover, it is considered to be an effective salvage fixation technique for failed traditional fixation or adjacent vertebral disease [9]. Compared to traditional pedicle screw fixation, the CBT technique is also less invasive, involving less soft tissue dissection, less blood loss, shorter operative time and shorter length of hospital stay [7].

However, the CBT technique has some limitations. In real surgeries, the posterior structures (mainly the spinous processes) are often considered to be a significant obstruction to screw placement in this trajectory, especially in the upper lumbar spine. Thus, partial resection of the spinous processes and supraspinous ligament is often required, which may cause damage to the posterior ligamentous complex participating in spinal stability. In addition, due to the special trajectory of the CBT screw, there remains a risk of screw penetration through the pedicle in patients with small pedicles, which can result in nerve injuries and unstable fixation. None of these situations should be overlooked during the operation.

Presently, although morphometric and biochemical studies have confirmed the feasibility of the CBT technique, there remains a lack of related reports on spinous process violation and screw penetration during the screw insertion process. Therefore, the purpose of this study was to evaluate the risk of the above complications in CBT screw fixation and provide a reference for the clinical application of this technique using three-dimensional (3D) screw insertion simulation software.

## Methods

### Inclusion and exclusion criteria

All lumbar spinal CT scans (containing L1–5) for either trauma evaluation or for preoperative surgical planning at our institution were retrospectively reviewed between January 2017 and June 2018. Patients with a lumbar fracture, compression of the vertebral body, ankylosing spondylitis, deformity, lumbar tumor or infection, or history of spinal surgery, were excluded. Patients with age of < 40 were excluded.

All CT scans were performed on a 16-slice CT scanner (Philips Brilliance 16; Philips Medical Systems, Eindhoven, the Netherlands). Scan parameters included 120 kV, 200 mA, a 512 × 512 matrix, a layer thickness of 1 mm, and a pitch of 1 mm.

### Computer simulation

**Fig. 1:**
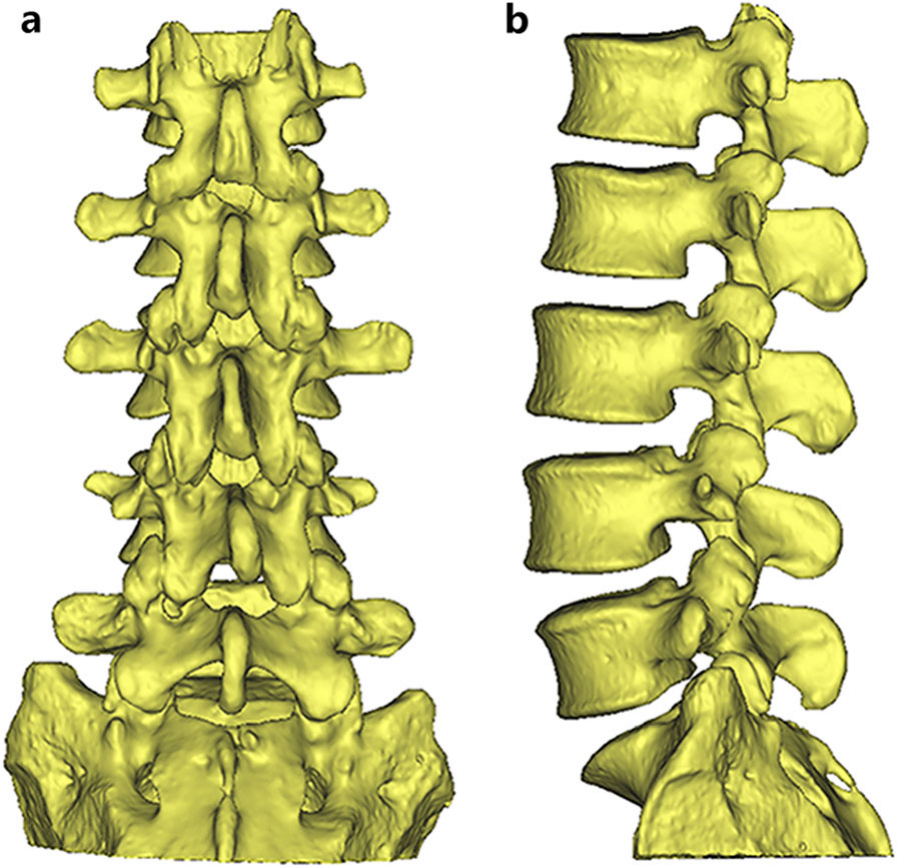
The anteroposterior (**a**) and lateral (**b**) view of the three-dimensional lumbar model

CT images of the lumbar spine were manipulated using Mimics software (ver. 18.0; Materialise, Leuven, Belgium) and underwent 3D reconstruction. After the removal of unnecessary anatomic structures, normal 3D lumbar models were obtained (Fig. 1). Then, CBT screw placement was simulated on each segment. The screw size and insertion method were bilaterally identical. The simulation was performed by a spine surgeon.

### Screw trajectories

**Fig. 2:**
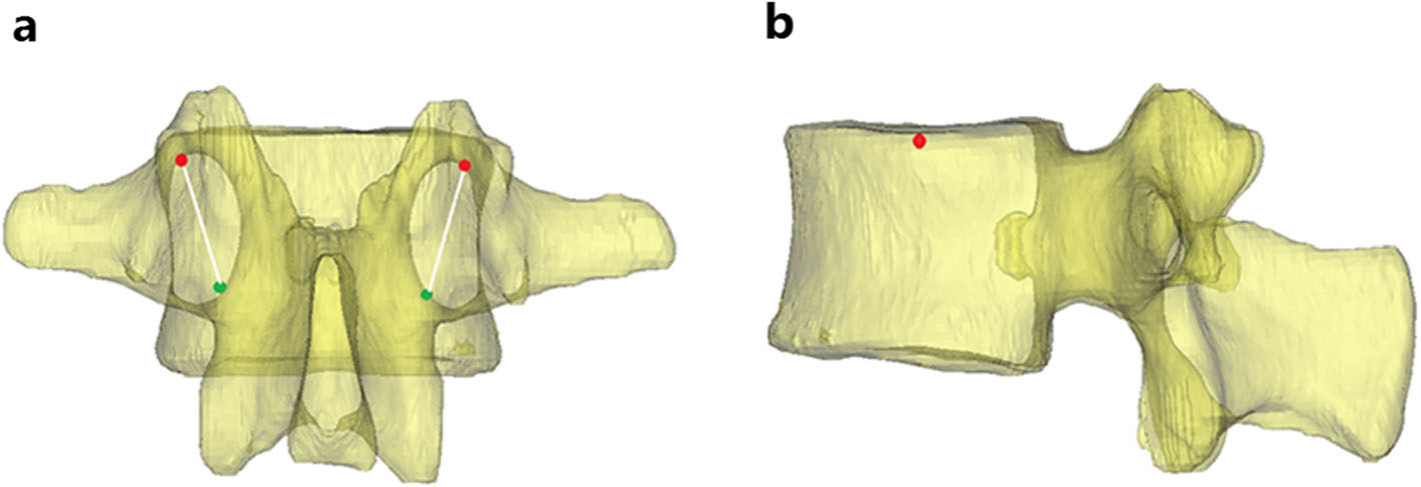
The entry points and trajectories were shown. In the anteroposterior view (**a**), the pedicle can be regarded as a clock face; the insertion was started at the 5 o’clock (green circle) position and aimed for 11 o’clock (red circle) orientation in the left pedicle, whereas insertion in the right pedicle began at the 7 o’clock (green circle) position and aimed for 1 o’clock (red circle) orientation. The endpoint (red circle) was set at the midpoint of the superior endplate without perforation in the lateral view (**b**)

CBT screw placement follows a medial-to-lateral path in the axial plane and a caudal-to-cephalad path in the sagittal plane through the pedicle, engaging maximally with the cortical bone from the pedicle to the vertebral body [4]. In this study, the target entry points and trajectories were based on the anteroposterior and lateral views of the 3D model. In the anteroposterior view, the pedicle can be regarded as a clock face, which can assist with intra-operative localization. The insertion was started at the 7 o’clock position and aimed for a 1 o’clock orientation in the right pedicle, whereas insertion in the left pedicle began at the 5 o’clock position and aimed for an 11 o’clock orientation in the anteroposterior view (Fig. 2a) [10, 11]. To create parity, the endpoint was set at the midpoint of the superior endplate without perforation in the lateral view (Fig. 2b). This trajectory was used for CBT screw placement in all lumbar levels.

### Screw dimensions

Currently, the 4.5 mm screw is commonly used in clinical surgery for CBT screw fixation. However, larger screws are also recommended for stronger fixation strength [12]. To provide a comparison among the various screw diameters, screws with a diameter of 4.5 mm, 5.0 mm, or 5.5 mm were used in the placement process in this study.

### Assessment of spinous process violation and screw penetration

**Fig. 3:**
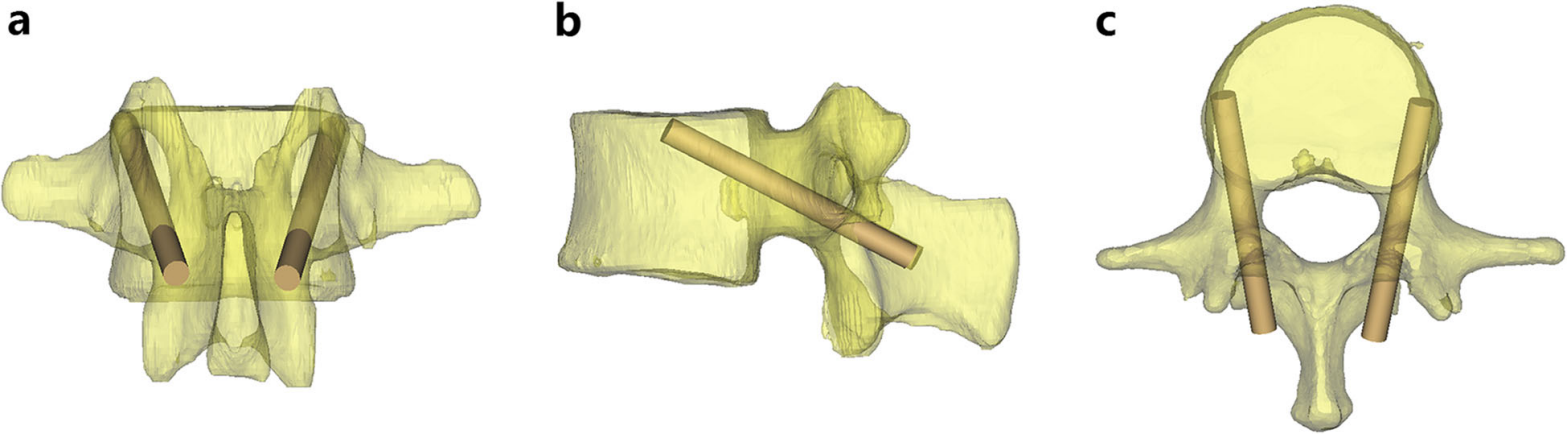
The illustration of successful CBT screw placement as shown on the coronal (**a**), sagittal (**b**) and axial (**c**) view

**Fig. 4:**
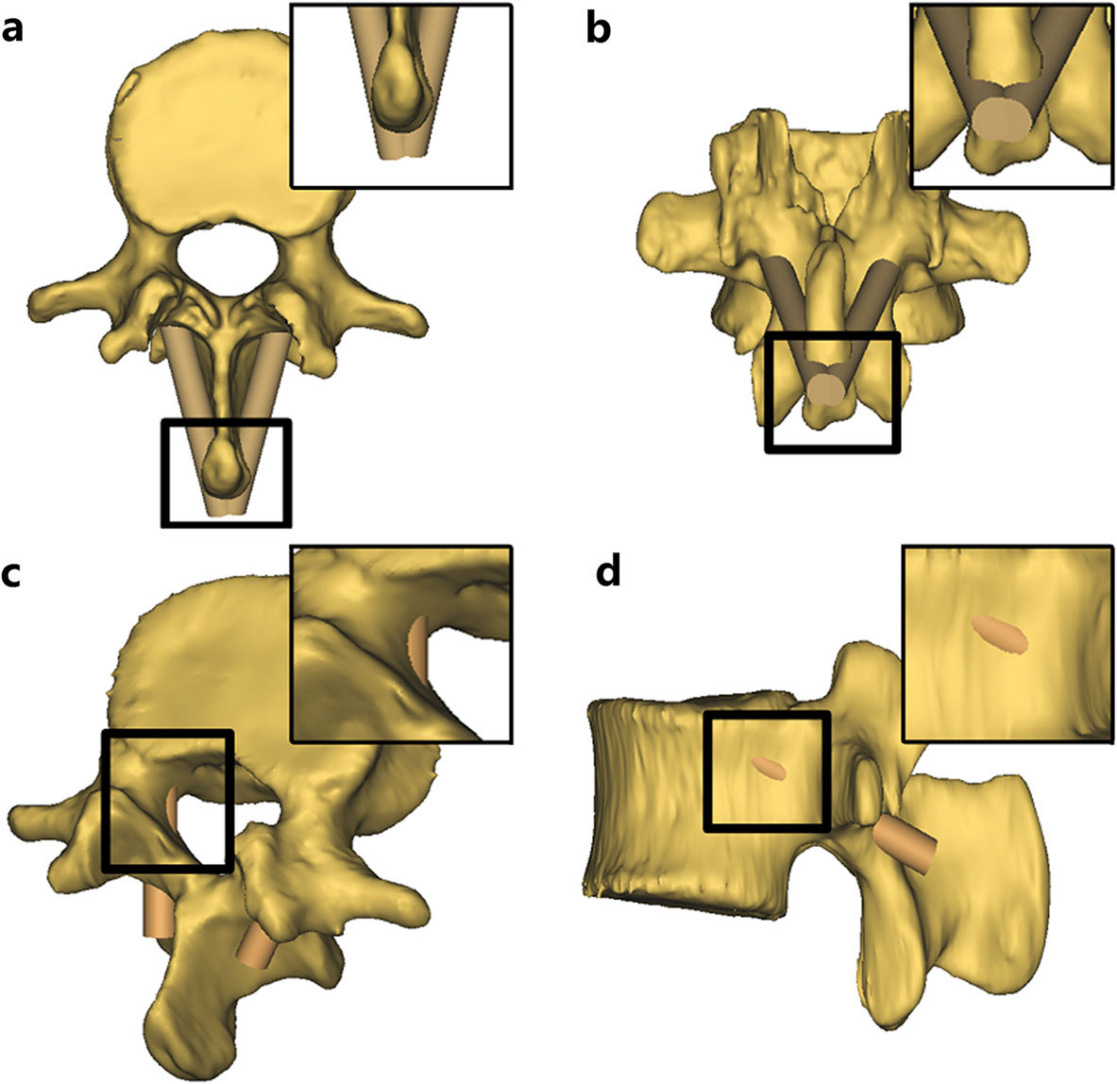
The illustration of complications in CBT screws placement. **a** and **b** show spinous process violation in axial and coronal views. **c** Screw penetration through the inner wall of the pedicle. **d** Screw penetration through the outer wall of the pedicle

Following the predetermined entry points and trajectories, CBT screw placement finished in L1–5 (Fig. 3). After screw placement, spinous process violation or screw penetration through the pedicle (through the inner and outer walls) could be directly observed in the 3D models (Fig. 4). Then, the incidence of the above complications was recorded and evaluated at each level. Each level was compared with all other levels. The incidence of each complication among different screw diameters, as well as by gender, was also compared. The assessment was performed by an independent spine surgeon.

### Statistical analyses

SPSS software (ver. 22.0; SPSS, Inc., Chicago, IL, USA) was used for all statistical analyses. The chi-squared test or Fisher’s exact test was utilized to compare the incidence of the above complications by gender, among the three screw diameters, and among the five levels. A *p*-value < 0.05 was considered to indicate statistical significance.

## Results

According to the selection criteria, 90 patients (48 males and 42 females, mean age: 63.7 years, range: 40–85 years) were enrolled in this study. All CT scans underwent 3D reconstruction and showed successful screw placement. Given the lack of any statistical difference between the two sides, one screw insertion was regarded as a separate study object. Thus, 900 screw insertions were contained in this simulation (180 screws in each segment).

Table 1 summarizes the incidence of spinous process violation and screw penetration through the pedicle (including the inner and outer walls) during the placement process. The difference among screw sizes, as well as lumbar levels, is shown in Table 2. The incidence of spinous process violation was observed in 68.3, 53.3, 25.5, 1.7, and 0% of segments from L1 to L5, respectively, using 4.5 mm screws. L1 and L2 were clearly associated with a particularly high rate of spinous process violation, whereas such violation barely occurred in L4 and L5. Similarly, 5.0 and 5.5 mm screws also showed a similar rate, and downward trend, of spinous process violation from L1 to L5. A significant difference was found among the five segments (*p* < 0.001); however, this appeared to be unconnected to gender (*p* > 0.05) or screw diameter (*p* > 0.05).

**Table 1:** The incidence of spinous process violation and pedicle wall penetration

Complication	L1 (mm)				L2 (mm)				L3 (mm)				L4 (mm)				L5 (mm)		
	4.5	5.0	5.5		4.5	5.0	5.5		4.5	5.0	5.5		4.5	5.0	5.5		4.5	5.0	5.5
A (%)																			
Male	62.5	64.6	65.6		52.1	52.1	53.1		25.0	26.0	26.0		0	0	0		0	0	0
Female	75.0	77.3	77.3		54.8	56.0	56.0		26.1	27.4	28.6		3.6	3.6	3.6		0	0	0
Total	68.3	70.5	71.1		53.3	53.8	54.4		25.5	26.7	27.2		1.7	1.7	1.7		0	0	0
*p* Value^a^	0.072	0.060	0.083		0.719	0.603	0.704		0.855	0.839	0.704		0.100	0.100	0.100		–	–	–
B (%)																			
Male	13.5	19.8	29.1		8.3	22.9	32.9		1.0	8.3	20.8		0	1.0	3.1		3.1	7.3	12.5
Female	20.2	32.1	42.8		17.8	29.7	36.9		4.8	17.8	27.4		2.4	6.0	10.7		10.7	16.6	21.4
Total	16.7	25.5	35.5		12.7	26.1	34.4		2.8	12.7	23.8		1.1	3.3	6.7		6.7	11.7	16.7
*p* Value^a^	0.229	0.058	0.056		0.056	0.297	0.516		0.186	0.056	0.304		0.216	0.099	0.069		0.069	0.051	0.066
C (%)																			
Male	2.1	4.2	14.5		0	1.0	9.4		0	0	0		0	0	0		0	0	0
Female	6.0	10.0	14.2		2.4	3.6	6.0		0	0	4.8		0	0	0		0	0	0
Total	3.9	6.7	14.4		1.1	2.2	7.8		0	0	2.2		0	0	0		0	0	0
*p* Value^a^	0.254	0.231	0.955		0.216	0.340	0.392		–	–	0.186		–	–	–		–	–	–

*A* Spinous process violation, *B* Screw penetration through the inner wall of pedicle, *C* Screw penetration through the outer wall of pedicle, *L* Lumbar

^a^ Comparison between genders

**Table 2:** The effect of lumbar level and screw size on three complications

Complication	Size (mm)	L1	L2	L3	L4	L5	*p* value^a^
A (%)	4.5	68.3	53.3	25.5	1.7	0	< 0.001
	5.0	70.5	53.8	26.7	1.7	0	< 0.001
	5.5	71.1	54.4	27.2	1.7	0	< 0.001
	*p* value^b^	0.831	0.971	0.936	1.000	–	
B (%)	4.5	16.7	12.7	2.8	1.1	6.7	< 0.001
	5.0	25.5	26.1	12.7	3.3	11.7	< 0.001
	5.5	35.5	34.4	23.8	6.7	16.7	< 0.001
	*p* value^b^	< 0.001	< 0.001	< 0.001	0.019	0.013	
C (%)	4.5	3.9	1.1	0	0	0	< 0.001
	5.0	6.7	2.2	0	0	0	< 0.001
	5.5	14.4	7.8	2.8	0	0	< 0.001
	*p* value^b^	0.001	0.002	0.018	–	–	

*A* Spinous process violation, *B* Screw penetration through the inner wall of pedicle, *C* Screw penetration through the outer wall of pedicle, *L* Lumbar

^a^ Comparison among lumbar levels

^b^ Comparisons among screw sizes

In general, the incidence of screw penetration through the pedicle was not high and differed between the inner and outer walls. The incidence of screw penetration through the inner wall with varying screw sizes (4.5, 5.0, or 5.5 mm) showed a downward trend from L1 to L4; in turn, L1 (16.7–35.5%), L2 (12.7–34.4%), L3 (2.8–23.8%) and L4 (1.1–6.7%). However, the incidence in L5 (6.7–16.7%) reversed this downward trend. As shown in Table 2, different levels showed a significantly different incidence of screw penetration through the inner wall (*p* < 0.001). Moreover, different screw sizes also resulted in an apparent difference in the incidence on L1-L5 (*p* < 0.05). In addition, screw penetration through the outer wall was rare compared to that through the inner wall, and did not tend to occur in the lower lumbar spine. The incidence of screw penetration through the outer wall also varied with screw size and lumbar level. Notably, there was no significant difference in screw penetration by gender.

## Discussion

The CBT screw fixation technique, as a modified fixation method for traditional pedicle screw fixation, has attracted increasing attention and has already been applied in clinical surgery [7, 8]. To achieve stronger internal fixation, the CBT screw follows a unique trajectory from the pedicle to the cortical bone surface, maximizing thread contact with the higher-density bone surface [4]. CBT screw fixation is minimally invasive and shows superiority in patients with severe osteoporosis. However, some potential complications cannot be completely avoided during the insertion process at particular trajectories. Spinous process violation and screw penetration through the pedicle are two of the most common complications and cannot be ignored operatively.

Given the lack of sufficient clinical data regarding these two complications, computer simulation software can be of great assistance in evaluating their incidence. Such software enables trajectories to be adjusted without breaking the specimen, and can test a large number of patients in visible models. Through 3D reconstruction of the lumbar spine and simulation of CBT screw placement, evaluation of the incidence of the two complications in each segment can be undertaken. Additionally, simulated placements are conducive to determining the entry point, insertion angle, and appropriate screw dimensions prior to a real operation, and help reduce the incidence of intraoperative complications.

In real CBT surgery, the unique trajectory of pedicle screw fixation often causes spinous process violation, where the posterior ligamentous complex can be a significant barrier to screw placement. To solve this problem, partial resection of the spinous process and supraspinous ligament is usually required before screw insertion, especially in upper lumbar surgery. Cheng et al. confirmed this in a cadaveric study [13]. They deemed the entry point to be close to the spinous process, which may lead to compression between the screw and the spinous process, as well as the lamina, without resecting the posterior element; this can ultimately result in the fracture of adjacent structures or trajectory deviations. In our study, the incidence of spinous process violation sequentially decreased from L1 to L5, which was in line with the gradual increase of lumbar vertebrae width from L1 to L5. In L1 and L2 in particular, the high rate of spinous process violation, of about 50–70%, indicated that partial resection of the posterior structure would be inevitable in most upper lumbar surgeries. In contrast, the low rate in the lower lumbar spine suggested superior maneuverability in the corresponding segments with this trajectory.

Screw penetration through the pedicle is among the common complications of pedicle screw fixation and carries the risk of neurovascular injury and pedicle fracture [14]. Through a systematic study of the anatomy of the lumbar pedicle, Li et al. [15] noted that the height of the pedicle is greater than its width, and that the upper and lower walls are formed of thicker cortical bone. Thus, screw penetration through the pedicle mainly occurs in the inner and outer walls rather than in the upper and lower walls. Compared to the traditional trajectory, the unique trajectory in this technique may increase the incidence of screw penetration during the placement process. Therefore, finer screws are usually applied in real CBT surgeries to reduce the incidence of screw penetration. Unlike the low incidence of screw penetration reported previously, screw penetration through the pedicle (including the inner and outer walls) clearly occurred in some cases in our study. The incidence of screw penetration was considered to be closely related to the morphology of the pedicle. As is already known, the morphology of the pedicle, including the shape and pedicle axis angle, differs by lumbar level [11, 16, 17]. With the increase in pedicle size from the upper to the lower lumbar spine, the incidence of screw penetration obviously decreased, suggesting the relative safety of this trajectory in the lower lumbar spine. Nevertheless, the risk of nerve injury and fixation instability should not be ignored, especially in the upper lumbar spine. Additionally, screw penetration through the outer wall is uncommon following use of the medial-to-lateral trajectory, and is much safer than penetration through the inner wall. Thus, screw penetration through the inner wall is more noteworthy.

Notably, the incidence of screw penetration through the L5 pedicle inner wall was higher than in L4, which was not consistent with the changes in lumbar pedicle size. The reasons for this may be as follows. First, the distinctive morphology of the L5 pedicle plays an essential role. The greater pedicle axial angle and deeper lateral recess compared to other levels increase the likelihood of screw penetration in L5 fixation [18]. Likewise, traditional trajectory pedicle screw fixation also has a certain rate of screw penetration through the inner wall despite the larger size of the L5 pedicle. However, contrary to the traditional trajectory, CBT does not follow the central axis of the pedicle and instead has a crossing angle with the pedicle. The greater the angle between the screw and the pedicle results in the higher the risk of screw penetration. Therefore, the high incidence of screw penetration in L5 fixation should be seriously considered.

It is generally accepted that screw purchase is positively related to screw diameter and length [12, 19]. A consensus has been reached that CBT demands finer and shorter screws than traditional techniques due to its special trajectory. However, it is usually considered that CBT could make up for the decrease of screw purchase caused by smaller screws. Currently, the 4.5 mm screw is recommended for use in clinical surgery for CBT fixation, to reduce the risk of screw penetration. In our study, the 5.0- and 5.5 mm screws also showed a relatively low incidence of screw penetration, especially in the lower lumbar spine. However, space for a safety margin around the screw in real surgery is required. The use of larger screws would reduce this space and increase the risk of pedicle fracture in real surgery. By investigating these concerns during the simulated operation, we determined that the space in the lower lumbar spine is adequate for placement of larger screws due to the larger size of the pedicle. Additionally, the incidence of screw penetration was not significantly related to screw size in the lower lumbar spine. Therefore, despite the higher incidence of screw penetration with larger screws, the use of larger screws (5.0 mm or 5.5 mm) is feasible in lower lumbar CBT fixation surgery to obtain stronger screw purchase. Nonetheless, surgeons should pay greater attention to fixation in L5 with larger screws because of the high rate of screw penetration. The preoperative measurement and evaluation of screw sizes can, to an extent, help reduce the risk of screw penetration [11, 20].

This study had some limitations. First, it was based on a computer simulation which is not as realistic as a cadaveric study. Second, there was no consideration of the size of the screw head and insertion tools, which inevitably led to an underestimation of the rate of spinous process violation. Third, the subjectivity of the operator may have a bias on the results. We were able to adjust the entry points and insertion angles several times before determining the correct trajectory for each case; this is not possible during an actual operation. Fourth, the relatively small sample size could be problematic in terms of statistical analysis. Finally, race, age, height, and weight were not considered in this study because of the restrictions of sample. In spite of these deficiencies, our study indicated the feasibility and safety of CBT screw fixation, especially in the lower lumbar spine.

## Conclusions

In conclusion, due to the high incidence of spinous process violation, there are more difficulties of CBT fixation in upper lumbar spine and risk of posterior element damage is higher. In addition, the low rate of screw penetration through the pedicle, with a screw diameter of 4.5 mm, suggests the safety of this method when carried out with small screws. Larger screws (5 mm or 5.5 mm) are more suitable for use in the lower lumbar spine under certain conditions, to provide stronger screw purchase. Moreover, CBT fixation in L5 deserves greater attention due to the unique morphology of the pedicle and its trajectory. In short, preoperative measurement and evaluation are of great importance in the choice of surgical trajectory or screw size before a real operation.

## Data Availability

All data generated or analyzed during this study are available upon reasonable request from the corresponding author.

## Abbreviations

CBT: Cortical bone trajectory
CT: Computed tomography
3D: Three-dimensional
